# Supplementary material for: Isolation and characterization of bovine herpes virus 5 (BoHV5) from cattle in India
Source: PLoS One. 2020 Apr 24;15(4):e0232093. doi: 10.1371/journal.pone.0232093 (PMC7182196; doi:10.1371/journal.pone.0232093)
Supplement: S1 Raw Image — (PDF) [file pone.0232093.s001.pdf]

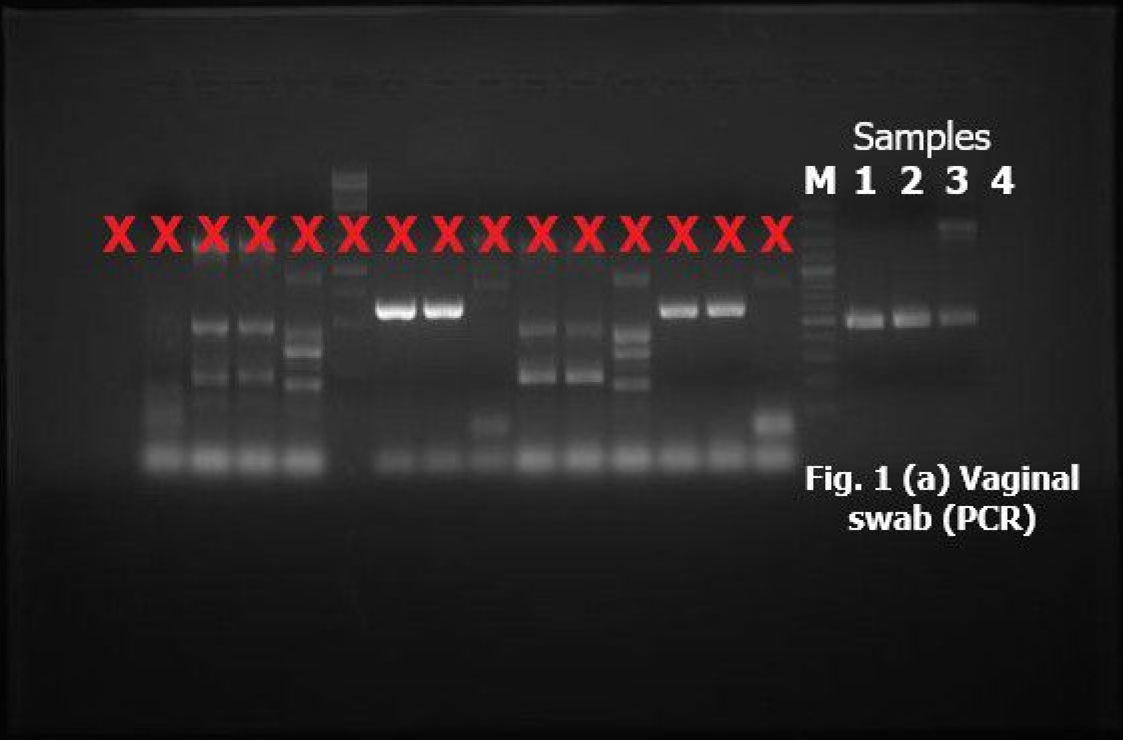

Fig. 1 (a) Vaginal swab (PCR)

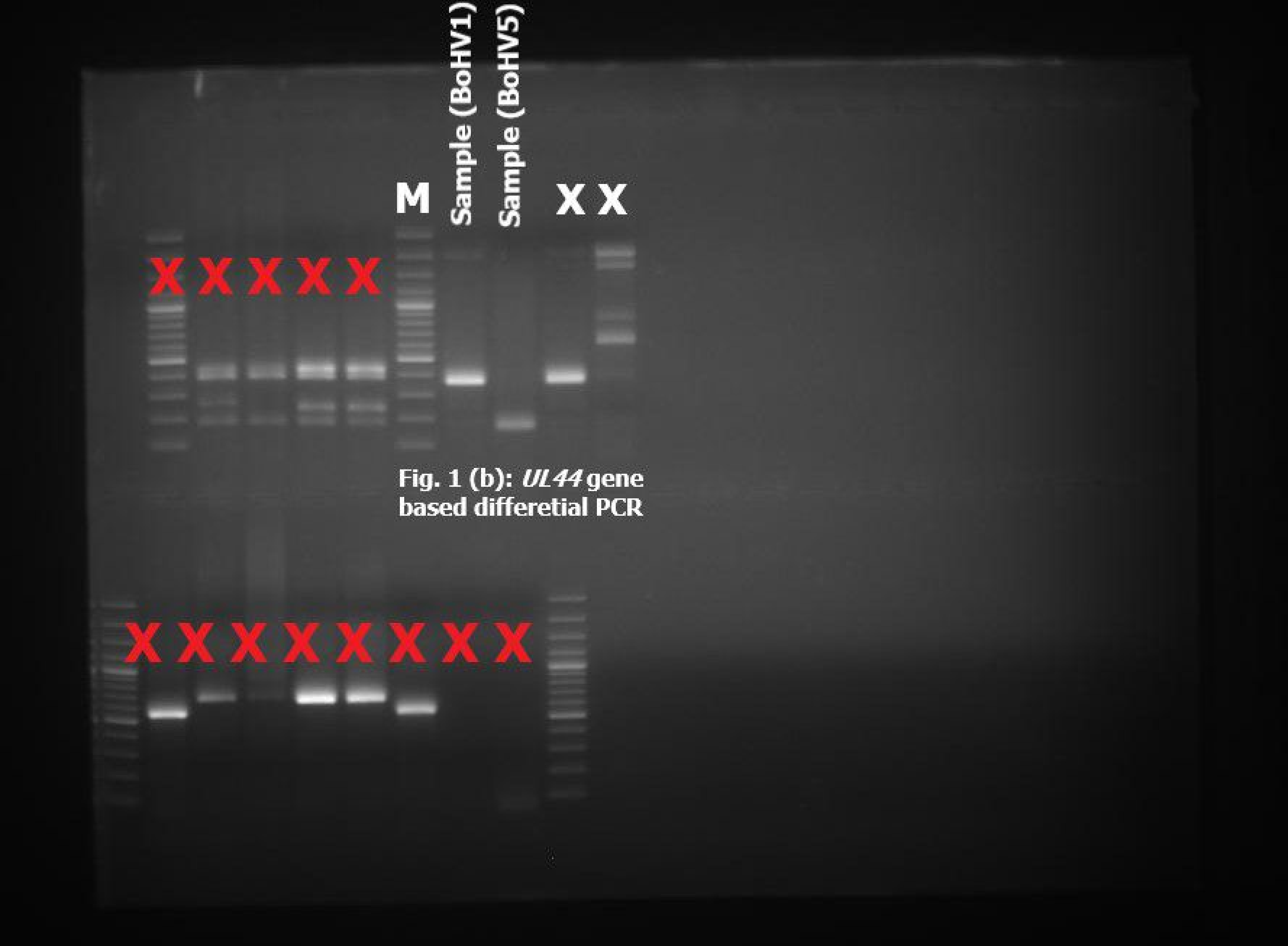

Fig. 1 (b): *UL44* gene based differetial PCR

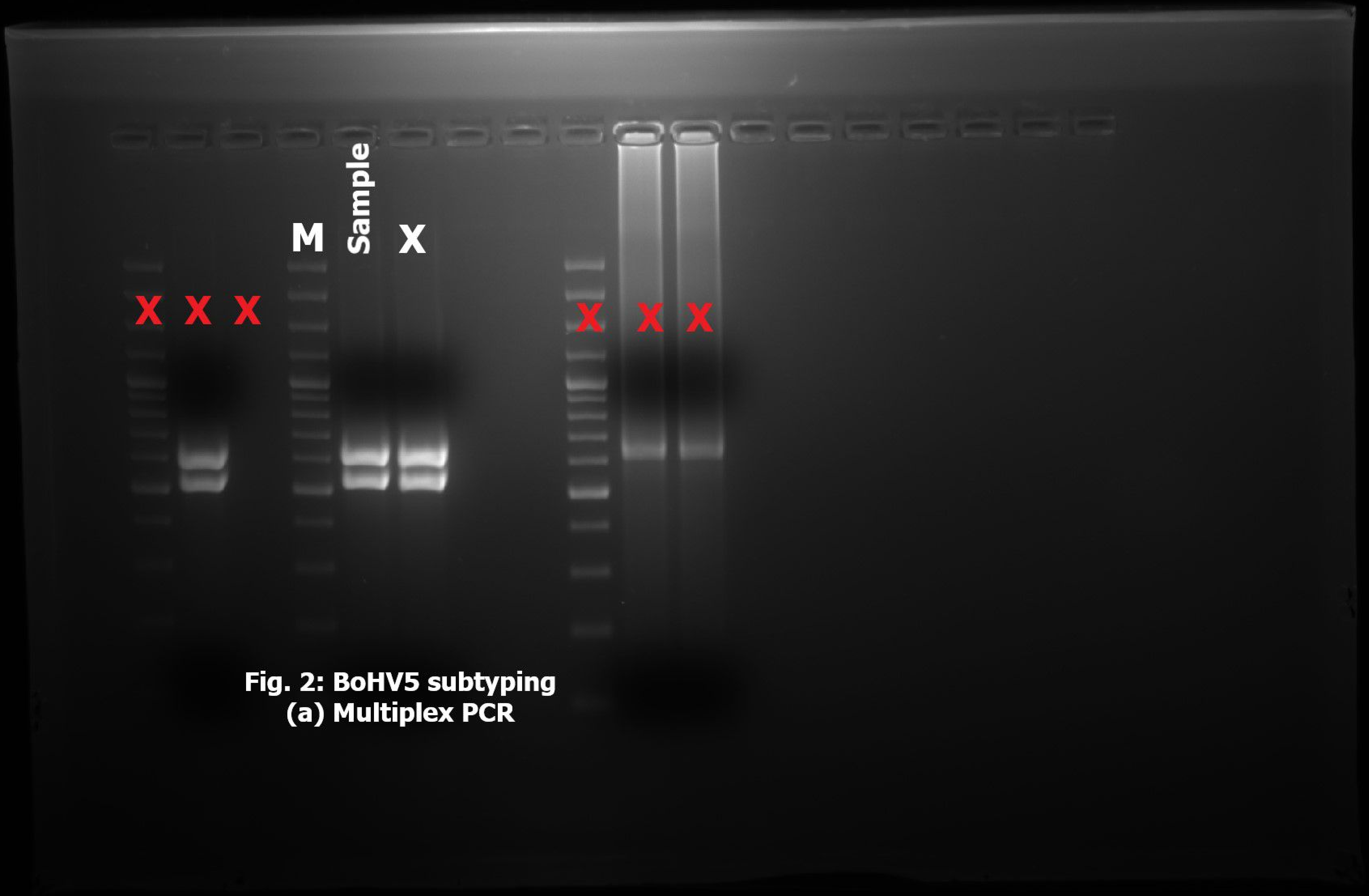

Fig. 2: BoHV5 subtyping
(a) Multiplex PCR

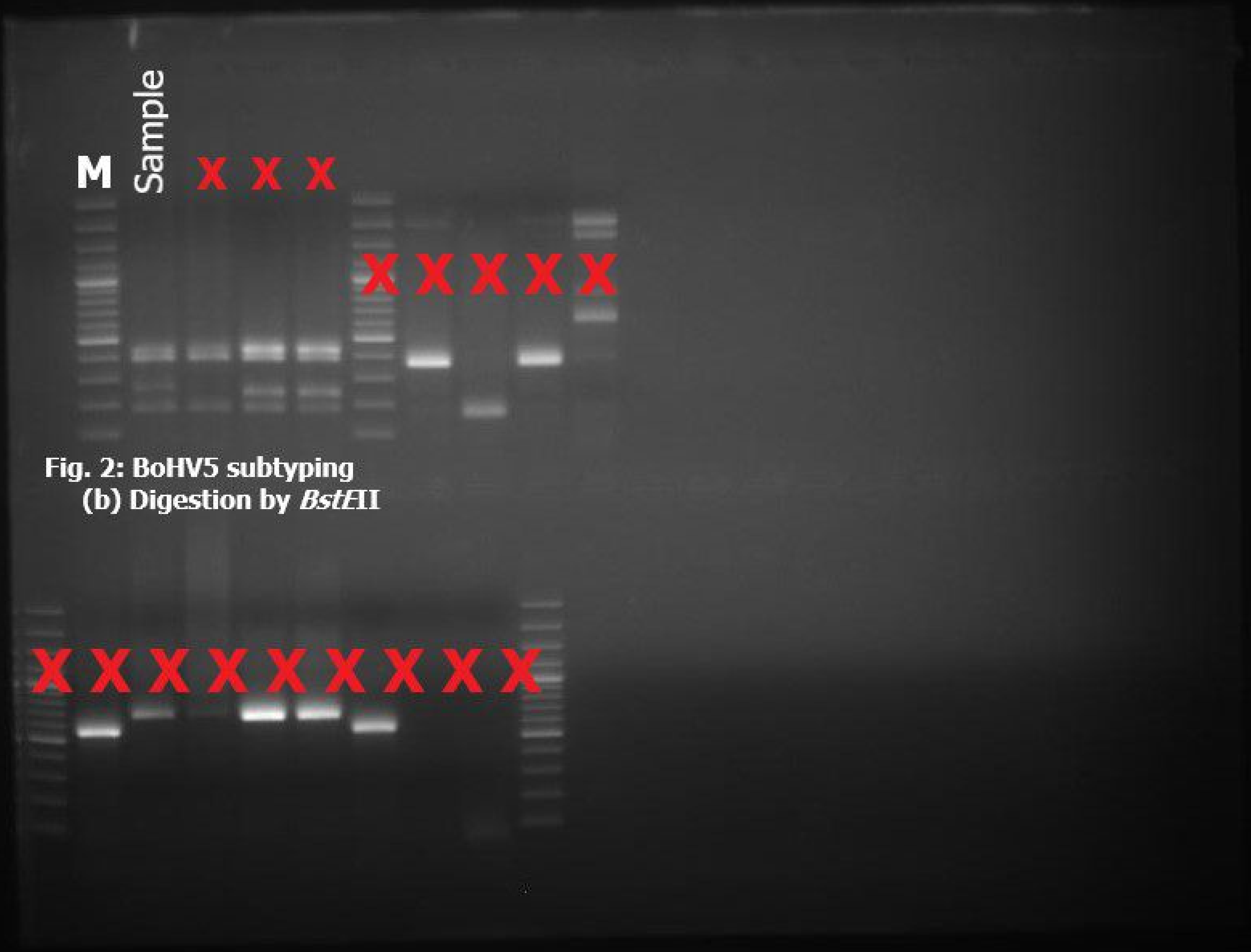

Fig. 2: BoHV5 subtyping
(b) Digestion by *BstE*II
