# Supplementary material for: Isolation and characterization of bovine herpes virus 5 (BoHV5) from cattle in India
Source: PLoS One. 2020 Apr 24;15(4):e0232093. doi: 10.1371/journal.pone.0232093 (PMC7182196; doi:10.1371/journal.pone.0232093)
Supplement: Attachment — Submitted filename: PlosONEreview.pdf [file pone.0232093.s002.pdf]

The manuscript is really interesting since there are only few reports associating BoHV-5 with abortion in cattle. Thus, this finding is novel and extends the geographical distribution of the virus to regions were infections have not been frequently reported. Nevertheless, there are some major points that the authors should address to better understand the implication of BoHV-5 in the case reported.

It is suggested to use the current virus nomenclature abbreviation for referring to the bovine alphaherpesviruses….BoHV-1 and BoHV-5

Virus seroneutralization test. …The cells were observed daily for appearance of CPE for determination of the antibody titres….

The authors should indicate whether they followed the standard seroneutralization procedure in which the test is read at 72 h…..it is not clear whether the read out was performed at the time of CPE appearance or when indicated by the control back titration at 72 h

Virus isolation. It is not clear from how many vaginal swabs the virus was isolated. It would be interesting to add a column in table 2 indicating isolation +or -

This point is important since Brucella is also a cause of abortion in cattle and only detection of BoHV5 genome is not indicative of virus involvement in the abortions.

Anti BoHV antibodies. According to the manuscript cattle in this study is not vaccinated against BoHV. However, since seroconversion is not demonstrated the presence of antibodies might indicate that cattle is latently infected, with infection occurring some time before the abortions. This should be indicated in the discussion.

Minor comments

Line 21. amplification of the viral genome in PCR….change IN PCR for BY PCR

Line 38. Bovine herpesvirus types -----Please change by current ICTV nomenclature…..bovine alphaherpesvirus types…

The following paragraph from lines 40 to 43 should be re-written. ….since IBR is the main respiratory disease it is characterized by conjunctivitis and rhinotracheitis….the genital infection is characterized by balanopostitis and infectious pustular vulvovaginitis and abortion ……..

40BHV1 is prevalent globally

41 and causes infectious bovine rhinotracheitis (IBR) in cattle which is characterized by

42 conjunctivitis, rhinotracheitis, balanopostitis, infectious pustular vulvovaginitis, enteritis,

43 abortion and sometimes encephalitis

Line 45….and mortality can causes up to 100%.....please change….and mortality can reach 100%

Line 171. ..been reported from India.   Please change to reported IN India
