# Supplementary material for: Isolation and characterization of bovine herpes virus 5 (BoHV5) from cattle in India
Source: PLoS One. 2020 Apr 24;15(4):e0232093. doi: 10.1371/journal.pone.0232093 (PMC7182196; doi:10.1371/journal.pone.0232093)
Supplement: Attachment — Submitted filename: Responses.doc [file pone.0232093.s003.doc]

**Reviewers response**

Reviewer #1: The manuscript is really interesting since there are only few reports associating BoHV-5 with abortion in cattle. Thus, this finding is novel and extends the geographical distribution of the virus to regions were infections have not been frequently reported. Nevertheless, there are some major points that the authors should address to better understand the implication of BoHV-5 in the case reported.

***We thank the Reviewer for his/her enthusiasm towards this study***

It is suggested to use the current virus nomenclature abbreviation for referring to the bovine alphaherpesviruses….BoHV-1 and BoHV-5

***BHV1/BHV5 has been changed to BoHV1/BoHV5 in the entire manuscript***

Virus seroneutralization test. …The cells were observed daily for appearance of CPE for determination of the antibody titres….

The authors should indicate whether they followed the standard seroneutralization procedure in which the test is read at 72 h…..it is not clear whether the read out was performed at the time of CPE appearance or when indicated by the control back titration at 72 h

***The final readings were taken at 72 h-post infection. The same has been described in the revised manuscript (materials and methods section).***

Virus isolation. It is not clear from how many vaginal swabs the virus was isolated. It would be interesting to add a column in table 2 indicating isolation +or -
This point is important since Brucella is also a cause of abortion in cattle and only detection of BoHV5 genome is not indicative of virus involvement in the abortions.

Anti BoHV antibodies. According to the manuscript cattle in this study is not vaccinated against BoHV. However, since seroconversion is not demonstrated the presence of antibodies might indicate that cattle is latently infected, with infection occurring some time before the abortions. This should be indicated in the discussion.

***Virus isolation was successful only in two out of three vaginal swabs. The same has been mentioned in the text (results) as well as in Table 2 in the revised manuscript.***

Minor comments

Line 21. amplification of the viral genome in PCR….change IN PCR for BY PCR

***Changed as per the suggestion***

Line 38. Bovine herpesvirus types -----Please change by current ICTV nomenclature…..bovine alphaherpesvirus types…

***BHV1/BHV5 has been changed to BoHV1/BoHV5 in the entire manuscript***

The following paragraph from lines 40 to 43 should be re-written. ….since IBR is the main respiratory disease it is characterized by conjunctivitis and rhinotracheitis….the genital infection is characterized by balanopostitis and infectious pustular vulvovaginitis and abortion ……..

40BHV1 is prevalent globally

41 and causes infectious bovine rhinotracheitis (IBR) in cattle which is characterized by
42 conjunctivitis, rhinotracheitis, balanopostitis, infectious pustular vulvovaginitis, enteritis,

43 abortion and sometimes encephalitis

***Rewritten as per the suggestions***

Line 45….and mortality can causes up to 100%.....please change….and mortality can reach 100%

***Changed as per the suggestion***

Line 171. ..been reported from India. Please change to reported IN India

***Changed as per the suggestion***

**Reviewer #2:**

This is a well-written manuscript that presents new information about the presence of BHV-5 in India, and of its potential to cause abortions. This manuscript should be of interest to those virologist that are presently working with this virus.

***We thank the reviewer for his/her enthusiasm towards this study.***
